# Mechanics of composite hydrogels approaching phase separation

**DOI:** 10.1371/journal.pone.0211059

**Published:** 2019-01-25

**Authors:** Xiufeng Li, Wolf Rombouts, Jasper van der Gucht, Renko de Vries, Joshua A. Dijksman

**Affiliations:** Physical Chemistry and Soft Matter, Wageningen University and Research, Stippeneng 4, 6708WE Wageningen, the Netherlands; University of Vermont, UNITED STATES

## Abstract

For polymer-particle composites, limited thermodynamic compatibility of polymers and particles often leads to poor dispersal and agglomeration of the particles in the matrix, which negatively impacts the mechanics of composites. To study the impact of particle compatibility in polymer matrices on the mechanical properties of composites, we here study composite silica- protein based hydrogels. The polymer used is a previously studied telechelic protein-based polymer with end groups that form triple helices, and the particles are silica nanoparticles that only weakly associate with the polymer matrix. At 1mM protein polymer, up to 7% of silica nanoparticles can be embedded in the hydrogel. At higher concentrations the system phase separates. Oscillatory rheology shows that at high frequencies the particles strengthen the gels by acting as short-lived multivalent cross-links, while at low frequencies, the particles reduce the gel strength, presumably by sequestering part of the protein polymers in such a way that they can no longer contribute to the network strength. As is generally observed for polymer/particle composites, shear-induced polymer desorption from the particles leads to a viscous dissipation that strongly increases with increasing particle concentration. While linear rheological properties as function of particle concentration provide no signals for an approaching phase separation, this is very different for the non-linear rheology, especially fracture. Strain-at-break decreases rapidly with increasing particle concentration and vanishes as the phase boundary is approached, suggesting that the interfaces between regions of high and low particle densities in composites close to phase separation provide easy fracture planes.

## Introduction

Designing high performance composite materials is a modern challenge in a wide range of fields, from aerospace to biomedical engineering: it is still a huge challenge to design a desired macroscopic performance by constructing a composite “from the ground up” by constructing its microstructure [1–4]. A specific type of composite materials with many applications in bioengineering are composite hydrogels. Hydrogels are being studied for many biomedical applications, including tissue engineering [5], as scaffolds [6], contact lenses [7] and drug delivery systems [8]. Frequently, nanoparticles are added to such hydrogels, to modify either their biofunctionality or their mechanical properties: for example, adding silver nanoparticles makes PVA/gum acacia hydrogels antibacterial [9], and magnetic nanoparticles allow for hydrogels to be remotely controlled by magnetic fields [10]. Nanoparticles are also used to modulate the mechanical properties of hydrogels, since their presence often strongly influences the hydrogel stiffness [11], porosity [12]and fracture resistance [13], which are all crucial for many applications. For instance, cartilage substitutes need to be resistant to high compressive stresses but at the same time have have tunable stiffness [14]. Also, when used as scaffold, the mechanical properties of the hydrogels strongly influence the biological response of the encapsulated or attached cells [15].

**Fig 1:**
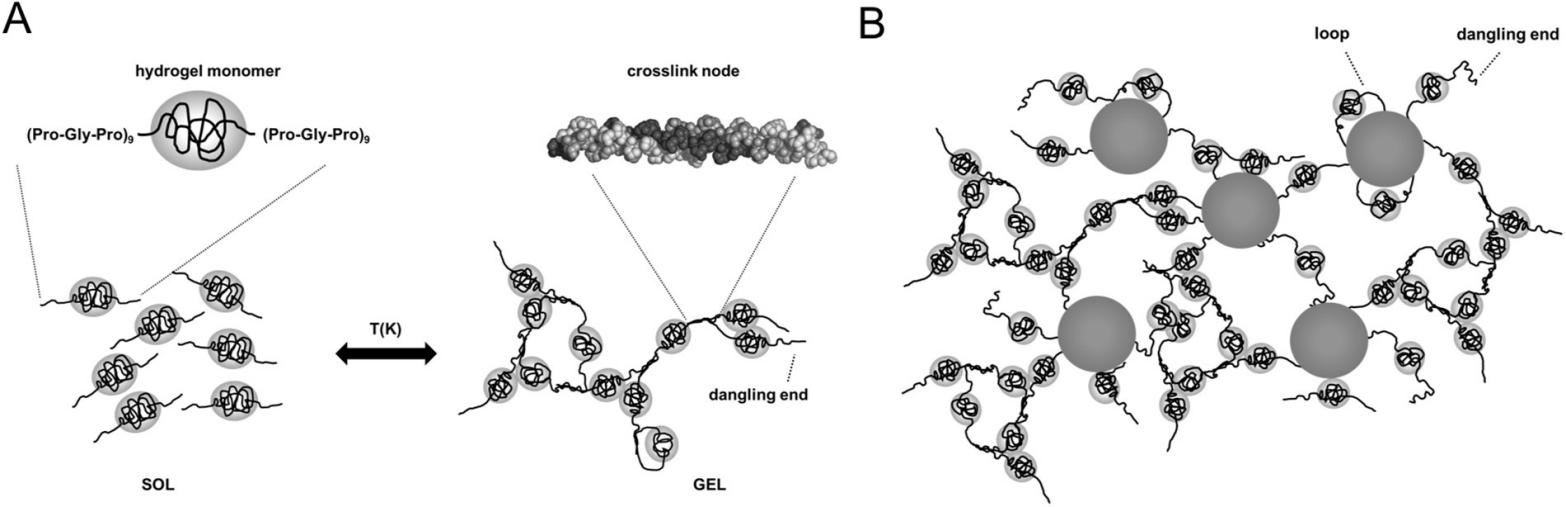
Model system for self-healing composite hydrogel. A: Thermogelation is caused by triple helix formation leading to trifunctional crosslinks. B: A schematic of the composite gel; all elements are roughly to scale.

In many (but not all) cases a highly desirable property of hydrogels and composite hydrogels for biomedical applications is that they are also self-healing so that they can, for example, be injected, or can adapt to changes in their environment. As a model biocompatible, and self-healing hydrogel, we have previously studied a telechelic protein-based polymer with a collagen-inspired amino-acid sequence abbreviated as ***T***_**9**_-***C***^**R**^_**4**_-***T***_**9**_. Its endblocks, ***T***_**9**_ = (PGP)_9_, are inspired by natural collagen, form triple helices at low temperatures. The midblock ***C***^**R**^_**4**_ is 399 amino acids long and does not contribute to the formation of helices over a wide range of solution conditions [16]. See Fig 1.

The self-healing hydrogels formed by this protein-based polymer have precisely defined architectures and melting trajectories. Their linear mechanical behavior, but also their non-linear mechanical properties and fracture strength can be precisely understood in terms of the known behavior of the triple helical end blocks and random coil midblocks [17, 18]. The well-defined nature of the protein-based polymers, and the fact that the mechanical behavior of the hydrogels they form are so well understood, makes this an ideal system to develop a more detailed understanding of composite self-healing hydrogels that incorporate nanoparticles.

With this in mind, we here study the effect of filling the ***T***_**9**_-***C***^**R**^_**4**_-***T***_**9**_ hydrogels with increasing amounts of nanoparticle fillers. As model nanoparticles we use SiO_2_ nanoparticles with a radius of about 20nm (Ludox). Such SiO_2_ nanoparticles are widely available and have good biocompatibility, making them good model particles for our purpose. The polypeptides feature a wide range of chemical groups that can potentially have weak interactions with the silica surface, so we expect the particles to be weakly interacting with the hydrogel matrix. This is probably typically the case for hydrogels where nanoparticles are added to endow hydrogels with extra functionalities such as antimicrobial activity [9], but not when the main purpose of the nanoparticle fillers is to act as multivalent physical crosslinks for mechanically reinforcement [19], since in that case polymer-nanoparticle interactions are deliberately engineered to be very strong instead.

For polymer-particle mixtures, when particles have unfavorable interactions with the polymers, phase separation will occur beyond some particle concentration. For particle-polymer composites, poor compatibility of particles and polymers leads to difficulties in dispersing the particles in the polymer matrix, and to particle agglomeration in the matrix [20]. Segregation of polymer and particles sometimes can be opposed by strongly attractive polymer-particle interactions (phase separation could still occur through bridging flocculation), but for the fairly typical case of weakly attractive interactions between polymers and particles it can often not be prevented completely. Our composite is thus a perfect model system for studying in detail how particle-polymer segregation affects the mechanical properties of composite hydrogels in the limit of weakly attractive interactions between the particles and the polymer matrix.

The model system is illustrated in Fig 1. The ***T***_**9**_ triple helix is around 8nm long, and the radius of the ***C***^**R**^_**4**_ polymer coil is around 7nm [16]. At temperatures above the melting point of the ***T***_**9**_ triple helices (around 37°C for the concentrations used to make hydrogels) the ***T***_**9**_-***C***^**R**^_**4**_-***T***_**9**_ polymers exist as free polymer coils (Fig 1A). Below the melting point, triple helices form. A small fraction of the polymers forms elastically inactive loops, but most polymers are elastically active [17] (Fig 1B). Various weak interactions determine the structure of the self-healing composite hydrogels, as illustrated in Fig 1B: ***T***_**9**_ blocks may form triple helices or adhere to the surface of the SiO_2_ particles. The ***C***^**R**^_**4**_ blocks may also weakly adhere to the surface of the SiO_2_ particles. Finally, in the bulk solution, structure formation is dominated by the formation of ***T***_**9**_ triple helices. Elastically inactive loops not only occur in the bulk, but may also occur at the surface. We study the macrostructural behavior and mechanical properties of the composite hydrogel as a function of nanoparticle concentration with rheology. We probe network formation, frequency dependence of storage and loss moduli, fracture dynamics and recovery in a single rheological protocol for each sample.

As expected, our model composite hydrogels do indeed show phase separation beyond a critical concentration of added SiO_2_ particles, and we probe the mechanical properties of the hydrogels over the entire one-phase region. Due to the well-defined nature of the system we can separate relaxations due to breakage of the triple-helices [18]from relaxations due to breakage of the much weaker bonds between the polymers and the surface, and such a separation is useful for developing understanding of the mechanics of self-healing composite hydrogels. Most importantly, for our model system we can show in detail how the approach to phase separation correlates with characteristic changes in the mechanical properties of the composites.

## Materials and methods

### Recombinant protein-polymers

Production and purification of the recombinant protein-polymers ***T***_**9**_-***C***^**R**^_**4**_-***T***_**9**_ and ***C***^**R**^_**4**_ was performed as described before [16, 21].

### Light scattering

A Zetasizer NanoZS apparatus (Malvern Instruments, UK) equipped with a 4mW He-Ne ion laser (λ = 633nm) was used to perform dynamic light scattering measurements. A stock solution of 0.1wt% SiO_2_ was prepared by diluting 50wt% LUDOX TM-50 (pH 9) in a prepared 50mL phosphate buffer (I = 10mM, pH 7), leading to a final pH around 7. The phosphate buffer was filtered before use (0.2μm filters). Given amounts of ***T***_**9**_-***C***^**R**^_**4**_-***T***_**9**_ and ***C***^**R**^_**4**_ proteins (0-2.5mg) were dissolved in 1mL stock solutions, which were left to equilibrate for 1h at 40°C in a thermo-heater with a shaking function before measurements. The intensity fluctuations of light scattered by particles were determined from an average of three autocorrelation measurements carried out at 40°C using a scattering angle of 173°. The hydrodynamic radius was obtained using standard light scattering theory.

### Preparation of composite hydrogels

Hydrogels studied here always consisted of 1mM ***T***_**9**_-***C***^**R**^_**4**_-***T***_**9**_ protein-polymer and varying volume fractions (0-7%) of SiO_2_ nanoparticles at pH 7. To prepare the composite hydrogels, 12.59mg ***T***_**9**_-***C***^**R**^_**4**_-***T***_**9**_ protein-polymer was always dissolved in different amounts of phosphate buffer (I = 10mM, pH 7) (300μL, 280.32μL, 266.44μL, 247.50μL, 229.40μL, 223.08μL). Next, 50wt% LUDOX (pH≈9) was adjusted to pH 7 by titration with 1M HCl. Different amounts of this LUDOX dispersion (0μL, 19.68μL, 35.56μL, 52.50μL, 70.60μL, 76.92μL) were added into the protein solutions. The total volume of all samples was 300μL. All samples were prepared in the same way and left in a thermo-heater with a shaking function at 50°C for 1 hour to reach an equilibrium in which no triple helices are present. Note that the pH slightly deviates from 7 after dissolving the protein; however, the pH change is small and since the protein concentration is constant, we assume that the pH changes will be the same for all samples.

### Turbidity measurement

The turbidity of all samples was quantitatively determined using a UV-Vis spectrophotometer (Thermo Scientific Evolution 220). Composite hydrogels were prepared in a quartz cuvette as described before. It was observed that during cooling the turbidity changed only during the first 30min. Therefore the turbidity of each sample was determined after a cooling period of more than 30min, by measuring the transmittance at a wavelength λ = 500nm.

### Cryo-SEM

The Cryo-SEM used here is a FEI Magellan 400 equipped with a Leica cold storage. Preparation of composite hydrogels was done as described before [16]. After preparation, the 1-4mm^3^ small samples were rapidly frozen in liquid nitrogen and then quickly fractured, removing the top part to obtain an open structure and allowing for imaging of the bulk of the broken gel. Next, samples were coated with a tungsten layer with a thickness around 20nm. Finally, samples were transferred to the Cryo-SEM.

### Rheology

Rheological measurements were performed with an Anton Paar MCR 301 or 501 rheometer equipped with a 1mL Couette geometry and Peltier element temperature control. A solvent trap with tetradecane was used to minimize evaporation of water. To minimize the sample volume, the lower part of the geometry, below the concentric gap, was filled with 725μL HT70 heavy oil (a perfluorinated polyether). We checked that this procedure did not affect rheological results. The viscous samples, equilibrated at 50°C were quickly transferred to the measurement geometry which was preheated at 50°C. After transferring the sample, the bob was lowered to the measurement position through the liquid samples. We then quenched the temperature to 20°C and the measurement was started. The following measurement protocol was used: (1) continuous oscillation measurement of the storage (G’) and loss (G”) modulus for 20h, at a fixed frequency and amplitude (*ω* = 6.28rad/s, *γ* = 1%) to follow gel formation. (2) a frequency sweep (*ω* = 0.01…100rad/s, *γ* = 1%) (3) a strain sweep (*ω* = 6.28rad/s, *γ* = 0.01…1000%). (4) continuous oscillation measurement of the storage (G’) and loss (G”) modulus for 8h, to follow recovery, at a fixed frequency and amplitude (*ω* = 6.28rad/s, *γ* = 1%). (5) After healing, we measure the shear stress at constant shear rate (0.1s^-1^) until a strain of 1000%. All data shown below are derived from this sequence of steps unless otherwise stated. We have done repeats of this protocol for different samples: 0% (1 time), 2% (1 time), 3.5% (3 times), 5% (1 time), 7% (2 times).

## Results and discussion

### Adsorption of *C*^R^_4_ and *T*_9_-*C*^R^_4_-*T*_9_ protein polymers onto surface of silica particles

**Fig 2:**
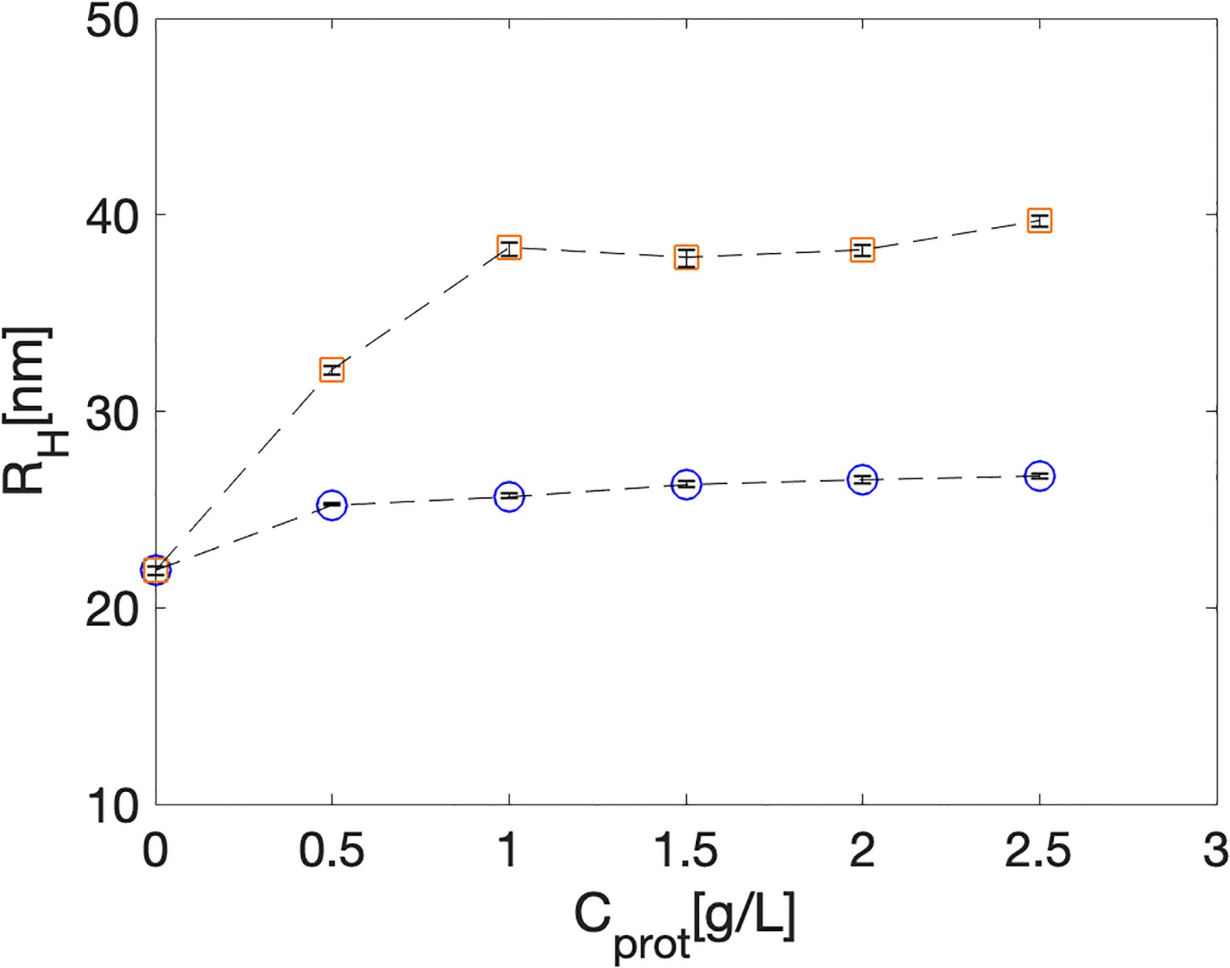
Dynamic light scattering of dilute solution of SiO_2_ nanopartices, to which increasing amounts of protein polymers *C*^R^_4_ and *T*_9_-*C*^R^_4_-*T*_9_ have been added. Hydrodynamic radius R_H_(nm) versus protein polymer concentration C_prot_(g/L). Orange squares: ***T*** **_9_-** ***C*** **^R^_4_-** ***T*** **_9_, blue circles**: ***C*** **^R^_4_**. Error bars indicate the range observed for three repeats.

First, we probe the association of individual silica nanoparticles with the protein polymers ***C***^**R**^_**4**_ and ***T***_**9**_-***C***^**R**^_**4**_-***T***_**9**_. To a dilute solution of silica nanoparticles (0.1wt%) we add increasing amounts of protein polymer, while remaining in the dilute limit of low protein polymer concentrations (<2.5g/L). Using dynamic light scattering, we monitor the increase in the hydrodynamic radius of the nanoparticles due to adsorption of the protein polymers at a temperature above the melting transition of the ***T***_**9**_ triple helices (40°C). Results are shown in Fig 2. Both polymers associate with the silica nanoparticles, as can be seen from the increase of the hydrodynamic radii R_H_ of the particles. The maximal increase for ***T***_**9**_-***C***^**R**^_**4**_-***T***_**9**_ is ΔR_H_≈18nm, whereas for ***C***^**R**^_**4**_, ΔR_H_≈5nm. Clearly the short ***T***_**9**_ blocks have a substantial influence on the adsorption which can only be explained if they have a higher affinity for the SiO_2_ than the ***C***^**R**^_**4**_ midblock.

### Composite gel formation

**Fig 3:**
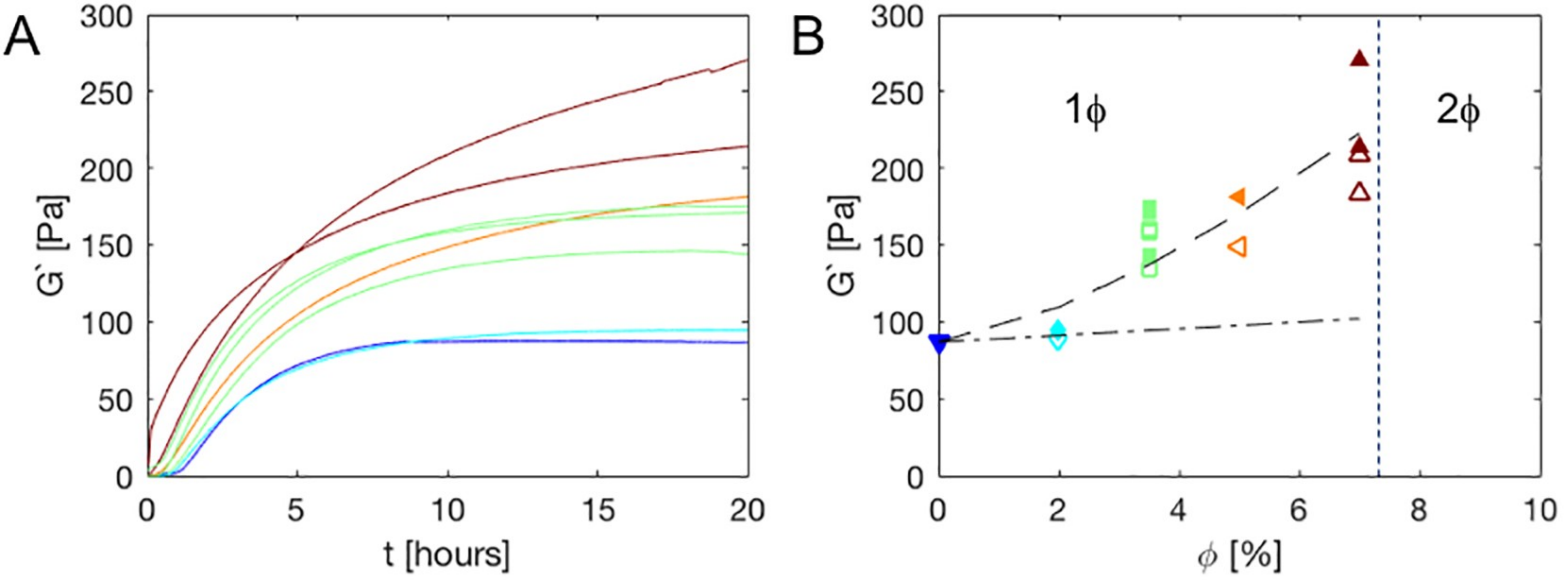
Composite gel formation and final moduli for 1mM *T*_9_-*C*^R^_4_-*T*_9_. A: Linear oscillatory rheology at fixed frequency and amplitude (*ω* = 6.28rad/s, *γ* = 1%) versus gelation time for different volume fractions of SiO_2_ nanoparticles. Dark blue: 0%, light blue: 2%, green: 3.5%, yellow: 5%, dark red: 7%. For 3.5% (green) and 7% (dark red) filler fraction, duplicates of the experiments are also given to illustrate the degree of reproducibility. B: The modulus after 10h (open symbols) and 20h (filled symbols) of gel formation; the color/symbol combination indicates the volume fraction of filler and is used for all figures, colors are as in A. The dash-dotted line indicates the linear Guth-Smallwood-Eshelby prediction (see text); the dashed line indicates a *ϕ*^3/2^ fit to indicate the nonlinear nature of the trend. The vertical dashed line indicates the boundary between the one phase (1*ϕ*) and two phase (2*ϕ*) regions.

The formation of 1mM ***T***_**9**_-***C***^**R**^_**4**_-***T***_**9**_ composite gels, after a temperature quench from T = 50°C to T = 20°C, was monitored using oscillatory rheology at a single low amplitude and frequency (*ω* = 6.28rad/s, *γ* = 1%). Results are shown in Fig 3. The formation of the network depends sensitively on the weight fraction of SiO_2_ nanoparticles: for 0 and 2% of SiO_2_ nanoparticles, we observe a sigmoidal growth of the gel modulus that reaches its plateau strength after about 10 hours—see Fig 3A. Higher nanoparticle weight fractions slow down the gelation dynamics, with the 7% gel clearly not yet having reached equilibrium even after 20h. A similar slow ageing was observed previously for particle-hydrogel composites, and ascribed to slow relaxation of non-equilibrium aggregated structures [22, 23]. Final moduli after 10h and 20h are shown in Fig 3B. A comparison with the classic Guth-Smallwood-Eshelby inclusion theory that would apply if the particles had been inert fillers [24–26]shows that the adsorption of the ***T***_**9**_-***C***^**R**^_**4**_-***T***_**9**_ to the nanoparticles leads to a significant increase of the modulus, beyond that expected for inert fillers.

**Fig 4:**
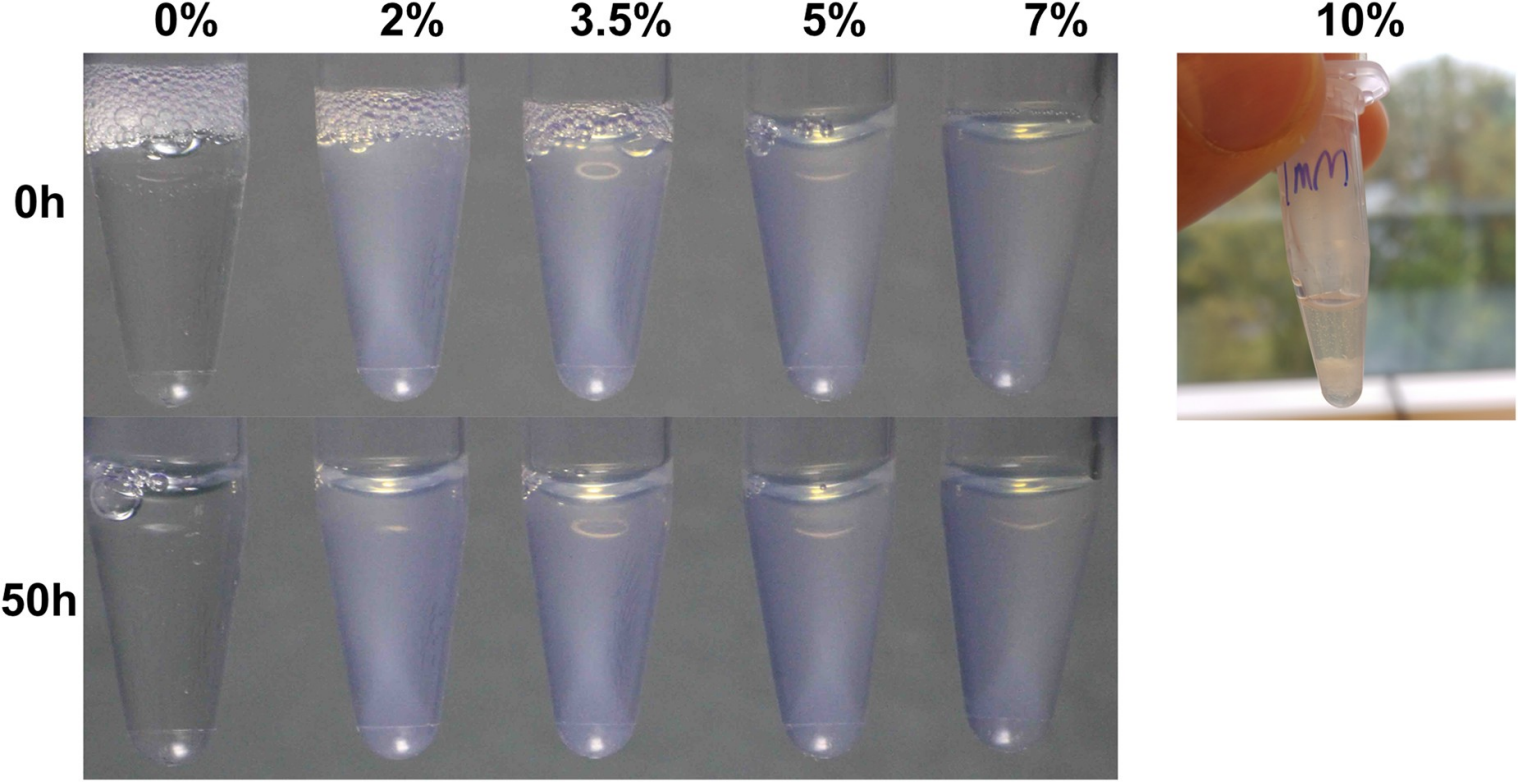
Limited compatibility of SiO_2_ nanoparticles with 1mM *T*_9_-*C*^R^_4_-*T*_9_. Pure protein sample is transparent. Composite samples (from 2% to 7%) are slightly turbid; no obvious phase separation occurs even after 50 hours. A composite sample with 10% particles phase separates a high temperature (50°C, right).

**Fig 5:**
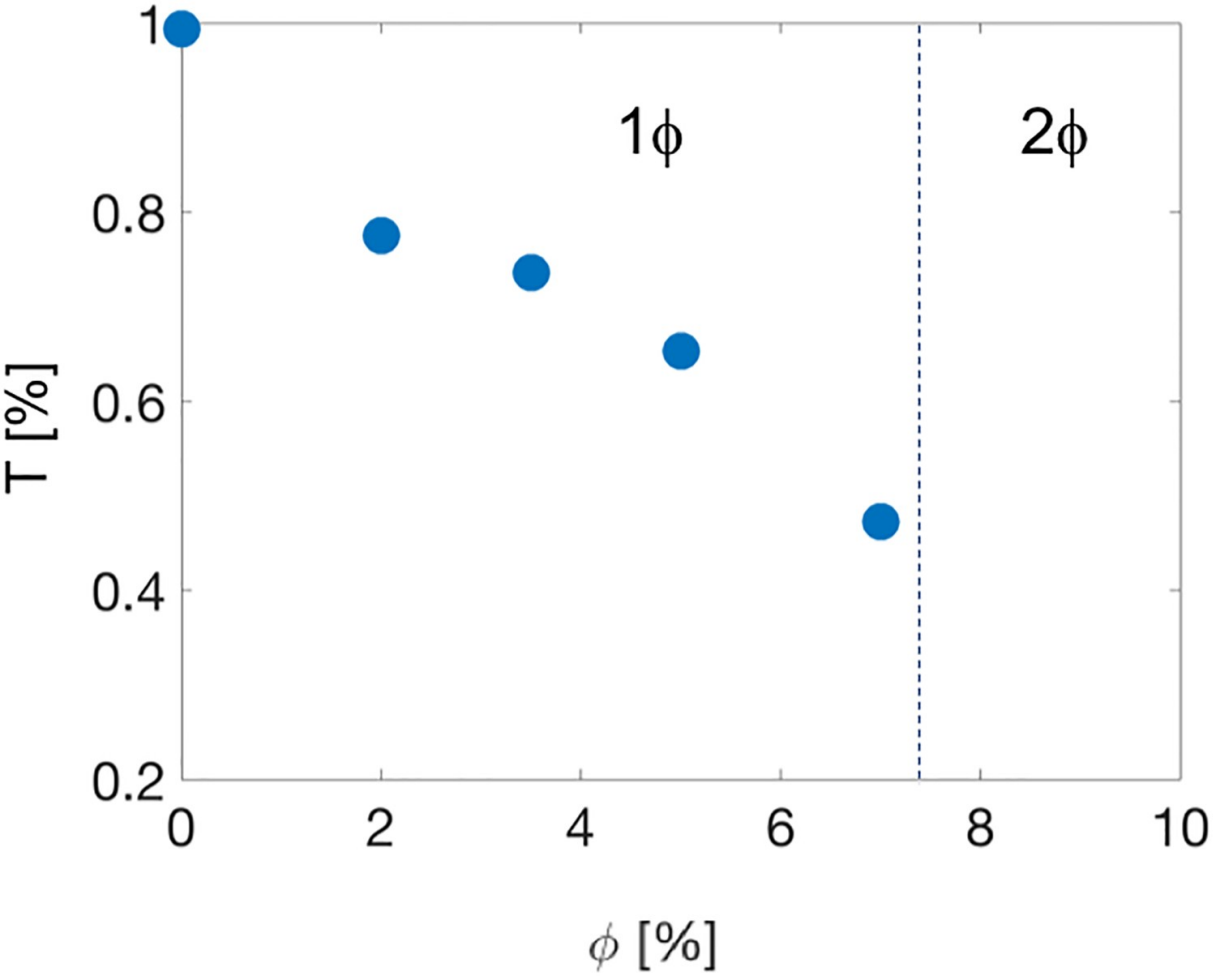
Transmittance of samples with various fractions of particles. The vertical dashed line indicates the boundary between the one phase (1*ϕ*) and two phase (2*ϕ*) regions.

As is the case for many polymer/nanoparticle composite systems in which the polymer/nanoparticle interactions are not very strong [27], there is only limited compatibility of the SiO_2_ nanoparticles and the 1mM ***T***_**9**_-***C***^**R**^_**4**_-***T***_**9**_ hydrogels. We need to distinguish the cases of compatibility at temperatures above and below the melting temperature of the ***T***_**9**_-***C***^**R**^_**4**_-***T***_**9**_ hydrogels (around 37°C) [16, 28]. Above the melting temperatures, systems are in a liquid state and presumably in thermodynamic equilibrium. We observe that at a temperature of 50°C and a protein concentration of 1mM, segregative phase separation occurs at particle volume fractions of 8% or larger. An example of a phase separated system at 10% of silica particles is shown in Fig 4. The phase boundary at high temperatures is not necessarily the same as that at low temperatures, but since macroscopic phase separation is usually slower than the gelation processes, the typical case is that any phase separation that will occur upon cooling is arrested at some stage by the gelation process. This also appears to be the case for the silica/***T***_**9**_-***C***^**R**^_**4**_-***T***_**9**_ composites: we observe a transmittance of cooled-down samples that strongly decreases with the nanoparticle concentration (Figs 4 and 5).

**Fig 6:**
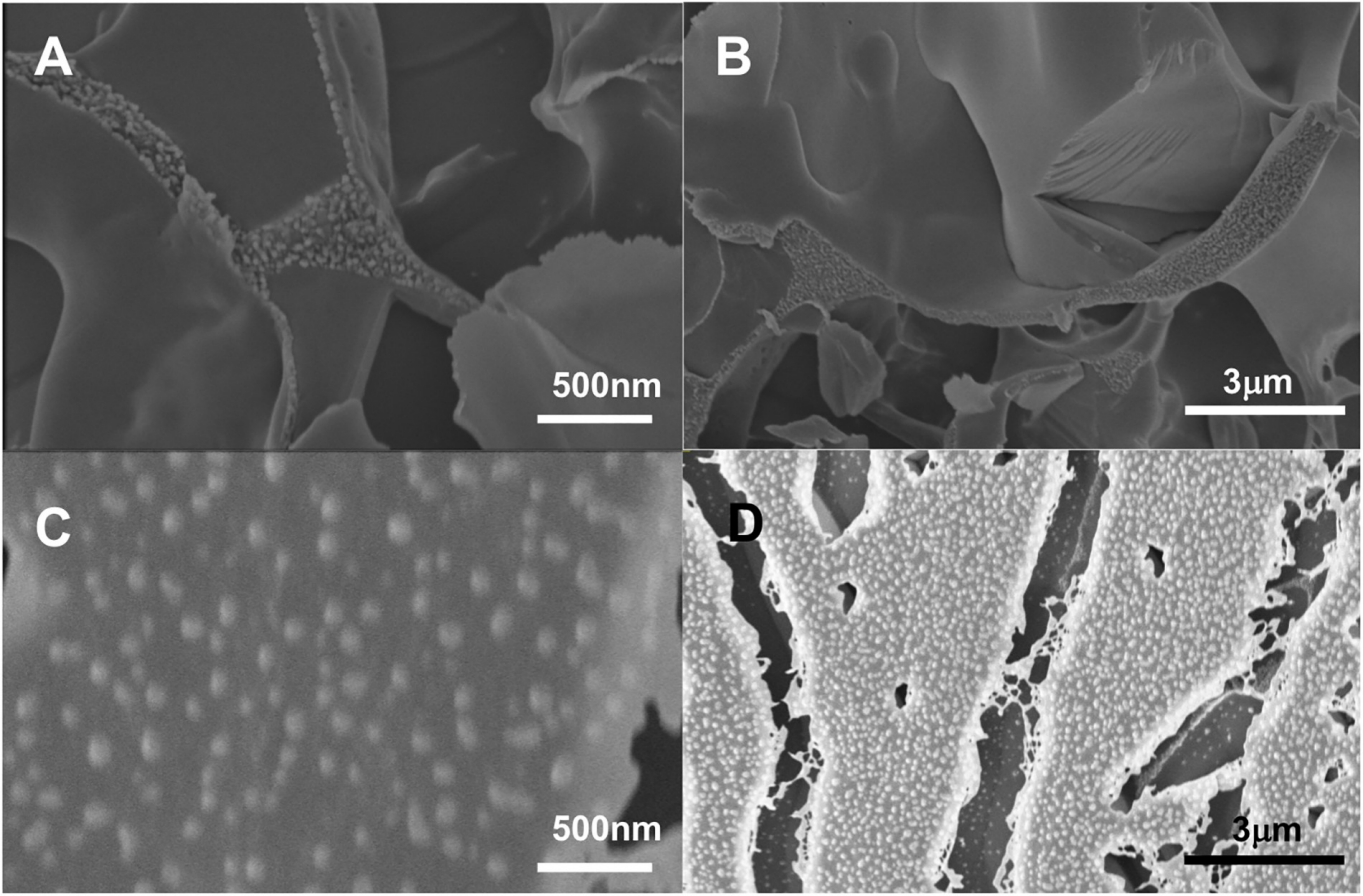
Cryo-SEM images. A and B are from a pure protein sample pictures, small dots are proteins. C and D are from a 7% composite sample. Large dots are SiO_2_ nanoparticles. Intermediate concentrations and full images are available in S1 Figs.

Cryo-SEM (Fig 6) reveals the macrostructure of the silica/***T***_**9**_-***C***^**R**^_**4**_-***T***_**9**_ composites. Control images for the pure protein networks (Fig 6A and 6B) allow us to distinguish the proteins from the much larger SiO_2_ nanoparticles. In a 7% composite sample (Fig 6C and 6D), the SiO_2_ nanoparticles appear to be well dispersed in the hydrogel matrix, suggesting that if during cooling a phase boundary was encountered, the resulting phase separation has not progressed far and was quickly arrested.

### Influence of particles on frequency dependent storage and loss moduli

**Fig 7:**
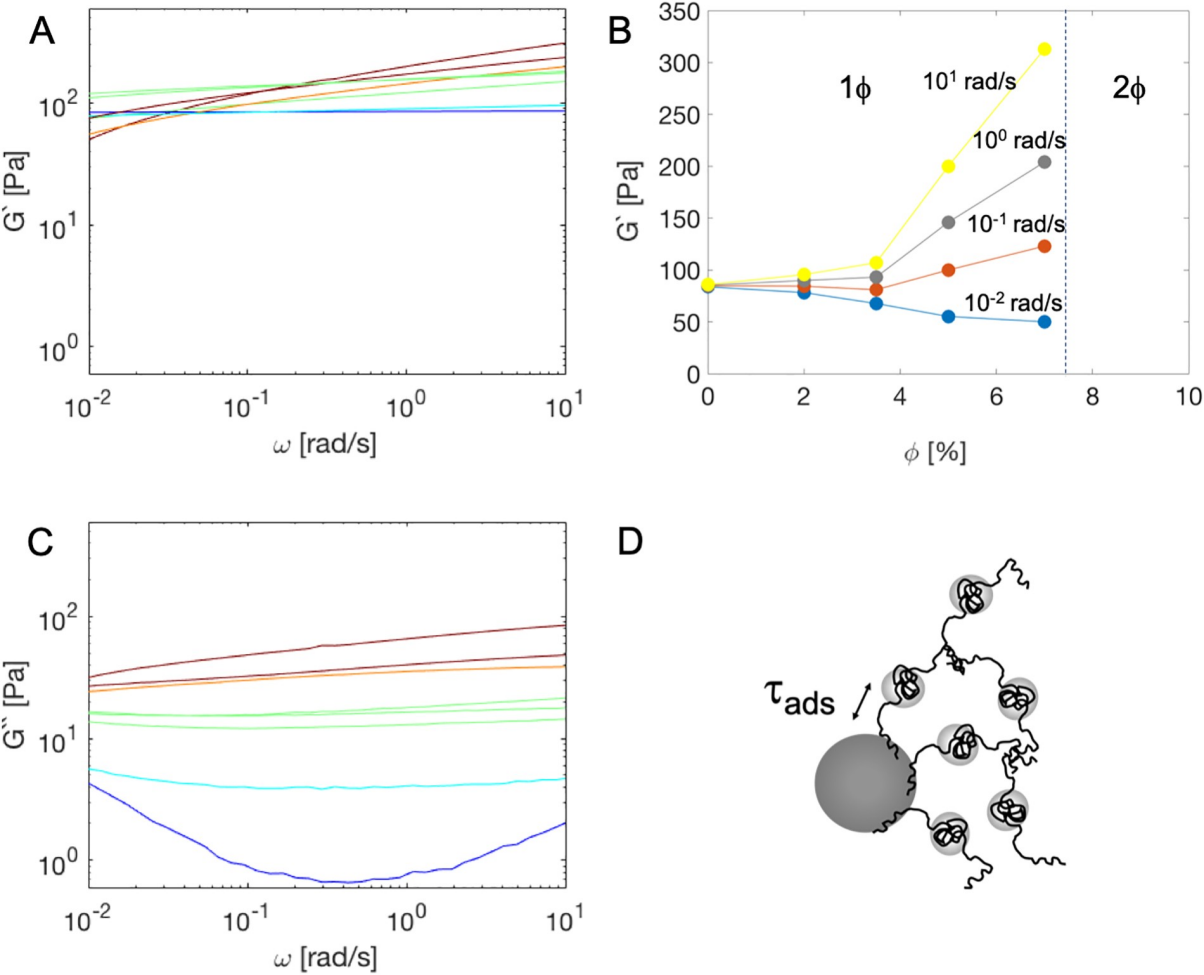
Frequency dependent storage and loss moduli G’(*ω*) and G”(*ω*) of 1mM *T*_9_-*C*^R^_4_-*T*_9_ composite hydrogels, at different particle concentrations. A: Storage modulus G’(*ω*) versus frequency *ω*. B: Storage modulus G’(*ω*) versus particle volume fraction [%], for a number of frequencies *ω*, as indicated. Also indicated is the phase boundary at 7.5%. C: Loss modulus G”(*ω*). Color coding is the same as in Fig 3. D: The average adsorption timescale *τ*_ads_ of the silica-protein binding is relevant for the frequency dependence of the rheology.

The frequency dependence of the storage and loss moduli of the composites after 20h is shown in Fig 7. In the absence of the nanoparticles, the storage modulus G’(*ω*) (Fig 7A) is frequency independent, as expected for a hydrogel for which the crosslinks (the triple helices) are essentially permanent over the frequency range that is probed [17]. Upon introducing the nanoparticles, the storage modulus G’(*ω*) becomes frequency dependent over the entire frequency range probed (10^-2^…10^2^rad/s), and this effect increases with increasing particle concentration. The nanoparticles affect the hydrogel mechanics in different ways at different frequencies: at very low frequencies (<10^-2^rad/s, extrapolating to lower frequencies), the nanoparticles soften the composite, whereas at higher frequencies, they stiffen it. This effect can be easily observed in Fig 7B, where we plot the storage moduli shown in Fig 7A as a function of volume fraction for a number of frequencies. We do not expect the extremely slow aging present in the 7% sample to affect the oscillatory tests in the current frequency window.

The frequency dependence must reflect the dynamics of the polymer adsorption on- and desorption off the SiO_2_ nanoparticles—see Fig 7D. The fact that the frequency dependence occurs over a wide range indicates that there is not a single characteristic frequency and timescale associated with polymer adsorption and desorption, but rather a broad frequency spectrum. The spectrum is so broad, in fact, that we are not able to reach frequency independent plateaus at either low or high frequencies. Although they have a very broad distribution of lifetimes, the polymer-particle bonds must of course have some average lifetime *τ*_ads_. At frequencies *ω* ≫ 1/*τ*_ads_ the particles act as multivalent physical crosslinks, increasing the strength of the network. For *ω* ≪ 1/*τ*_ads_ the nanoparticles no longer act as physical crosslinks and the storage modulus should tend to that of the bulk polymer network. The fact that we see the start of a reduction of the network strength at low frequencies, below that of the polymer network in the absence of the nanoparticles, means that polymer adsorption presumably has rendered some of the ***T***_**9**_-***C***^**R**^_**4**_-***T***_**9**_ polymers elastically inactive. This can occur for example, if the protein polymers adsorb with their two ***T***_**9**_ ends towards the surface of the nanoparticles. Since we cannot reach the low frequency plateau with oscillatory measurements, from the present data, we cannot yet estimate which fraction of protein polymers has become elastically inactive due to adsorption on the particles. However, the effect is significant: at *ω* = 10^-2^rad/s, the lowest frequency probed in the experiment, the storage modulus G’(*ω*) decreases from about 90Pa down to 50Pa when adding 7% SiO_2_ nanoparticles.

Next we consider the frequency-dependent loss modulus G”(*ω*) (Fig 7C). As reported before, without nanoparticles, the loss modulus is very low, but frequency dependent [17]. Adding the nanoparticles dramatically increases the loss modulus and reduces its frequency dependence. The frequency-dependence of the storage modulus already indicated that polymer adsorption and desorption occur at timescales corresponding to the entire frequency range probed in our experiment, hence the most obvious candidate dissipation mechanism that is proportional to the nanoparticle concentration is that of polymer desorption.

### Fracture and recovery

**Fig 8:**
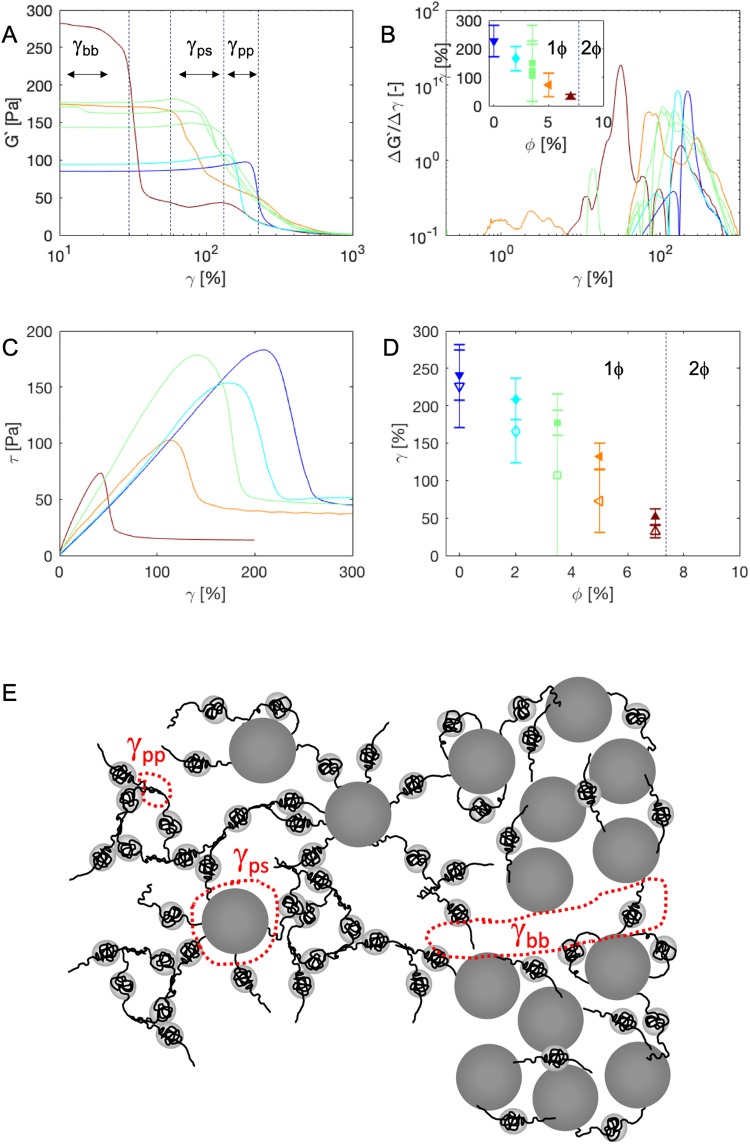
Fracture of 1mM *T*_9_-*C*^R^_4_-*T*_9_ composite hydrogels for a range of volume fractions of SiO_2_ nanoparticles. A: Storage modulus G’(*ω*) at *ω* = 6.28rad/s, as a function of strain *γ* for different fraction of SiO_2_ nanoparticles. Colors as in Fig 3A. B: The derivative Δ*G*′/Δ*γ* measuring the change in modulus in the strain amplitude sweep. Note the logarthmic scale on the vertical axis. The peak position represents the strain at break, the full width at half the maximum (FWHM) the error bar on the peak location. Inset: strain-at-break as a function of volume fraction of SiO_2_ nanoparticles taken from the strain sweep of A. Colors and symbols as in Fig 3B. C: Stress *τ* versus strain *γ* at a constant shear rate of $\dot{\gamma }=0.1{\text{s}}^{-1}$. D: strain-at-break as obtained from the flow curves in C, which we take as the fracture point (solid symbols). Open symbols are the fracture points obtained from the strain sweeps and as reproduced from B. The vertical dashed line indicates the boundary between the one phase (1*ϕ*) and two phase (2*ϕ*) regions. E: the different bond rupture mechanisms for protein-protein (pp) bond rupturing, protein-silica (ps) rupture and phase-separation induced boundary-boundary (bb) rupture as referred to in panel A.

The non-linear response of the composite hydrogels, in particular fracture, was characterized in two complementary ways: using a strain sweep at a fixed frequency (*ω* = 6.28rad/s) and by applying a linear increase in shear strain at a rate $\dot{\gamma }=0.1{\text{s}}^{-1}$ (measured after recovery for 8h from first strain sweeps). Results are shown in Fig 8. The nanoparticles dramatically decrease the fracture resistance of the hydrogels (Fig 8A and 8C), with the strain-at-break (determined by the largest derivative) vanishing as the phase boundary is approached (Fig 8B and 8D).

From the strain sweeps (Fig 8B) it appears that there are two distinct fracture processes. This is especially clear for the composite hydrogel with 7% SiO_2_ nanoparticles: a first fracture process catastrophically disintegrates a part of the network at a low strain of around 30%, we call this *γ*_*bb*_. After this, the remaining network shows some strain hardening and then fails at 150% strain (called *gamma*_*p*_ *p*), which is close to the strain-at-failure of the polymer hydrogel with no added nanoparticles. Presumably the first failure mainly involves breaking polymer-particle bonds between (almost) phase separated regions. For the failure at large strain, it is the polymer-polymer bonds that break, consistent with the fact that the strain-at-break for the second failure happens at around 150% strain, more or less independent of the volume fraction of added SiO_2_ nanoparticles. The strain-at-break decreases rapidly with increasing volume fraction of added SiO_2_ nanoparticles (Fig 8B), most likely due to the failure of individual polymer-silica bonds. We hence call the strains at which this happens *γ*_*p*_ *s*. A similar double-fracture behavior was reported for a silica-grafted double network hydrogel [29]. The constant stress at very large strains in Fig 8C is proportional to the effective viscosity of the fractured gel driven at a shear rate at $\dot{\gamma }=0.1{\text{s}}^{-1}$. Consistent with the linear oscillatory rheology, that showed a substantial increase in the loss modulus over all frequencies, we find that effective viscosity is lower as the gel “pulp” created by the easily induced fractures at higher particles concentrations make clumps smaller and yield more easily at a shear rate of $\dot{\gamma }=0.1{\text{s}}^{-1}$.

The observation that the strain-at-break (for the first failure) vanishes as the phase boundary is approached (Fig 8B inset and Fig 8D) suggests that the interfacial modulus for the phase separated system must be very low. As we approach phase separation, the interfaces between regions of high and low particle concentrations may provide very easy fracture planes. We clarify this perspective schematically in Fig 8E.

**Fig 9:**
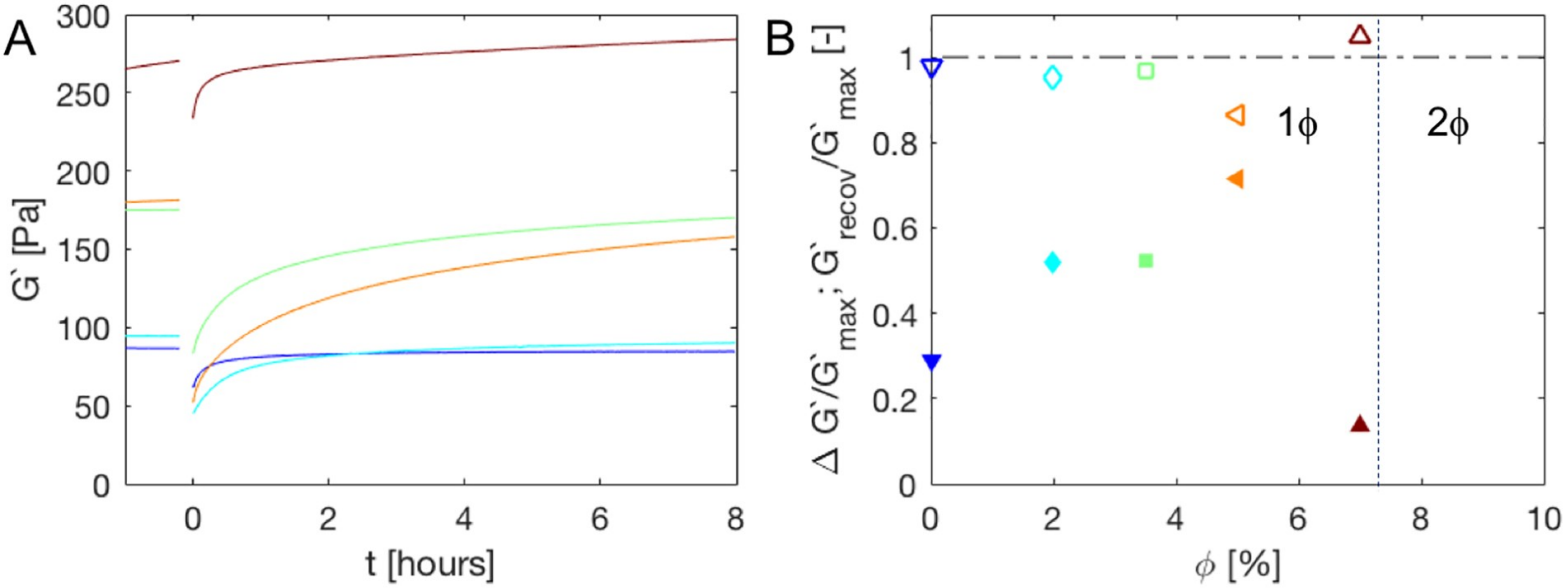
Recovery after strain sweep fracture as shown in Fig 8. For t<0, A: Show the last part of the formation dynamics as well (see Fig 3A), which ended about 4.5 hours before the recovery process started. The intervening time was used for frequency-dependent measurements and the strain sweep. B: The modulus reduction due to fracture (ΔG’/G’_max_, solid symbol) and eventual recovery ratio (G’_recov_/G’_max_, open symbol) as a function of filler fraction. For *ϕ* = 7% the recovery ratio is larger than one as the network had not reached equilibrium yet at the end of the formation period and throughout the experiment slowly continues its strengthening. The vertical dashed line indicates the boundary between the one phase (1*ϕ*) and two phase (2*ϕ*) regions.

Being held together completely by reversible interactions, we anticipate that after fracture, the composites should be able to mechanically heal. Indeed, as shown in Fig 9, recovery was observed immediately after the strain sweeps. Recovery after 8h was complete to within 80-95% of the strength last observed before fracture.

## Conclusions

We characterized the mechanical behaviour of a model self-healing composite hydrogel composed of a protein-based polymer with precisely defined architecture, mixed with silica nanoparticles. We observe that the addition of filler particles has profound consequences for the time dependent elastic and viscous response of the composite. At low frequency, the presence of nanoparticles reduces the elastic modulus of the composite; at high frequencies particles enhance the elastic modulus. The well-defined character of the composite ingredients gives us the ability to hypothesize that the low frequency modulus reduction is due to the nanoparticles sequestering polymer bonds, reducing the overall connectedness and thus strength of the gel network. The high frequency strengthening is most likely due to the nanoparticles acting as multivalent crosslinking node, strengthening the network. The tendency for nanoparticles to phase separate from the polymer has dramatic consequences for the fracture toughness of the composite: close to the phase separation boundary, the strain-at-break drops to zero, suggesting that, microscopically, fracture in phase separating composites is triggered in regions of high particle concentration. For all filler fractions, the composite retains its self-healing capacity, eventually restoring the elastic modulus even for the composite closest to the phase separation boundary.

## Supporting information

S1 Figs Cryo-SEM images. A: Pure protein sample. B: 2% composite sample. C: 5% composite sample. D: 7% composite sample.
(TIFF)
