# Supplementary figures and images for: Mechanics of composite hydrogels approaching phase separation
Source: PLoS One. 2019 Jan 25;14(1):e0211059. doi: 10.1371/journal.pone.0211059 (PMC6347237; doi:10.1371/journal.pone.0211059)

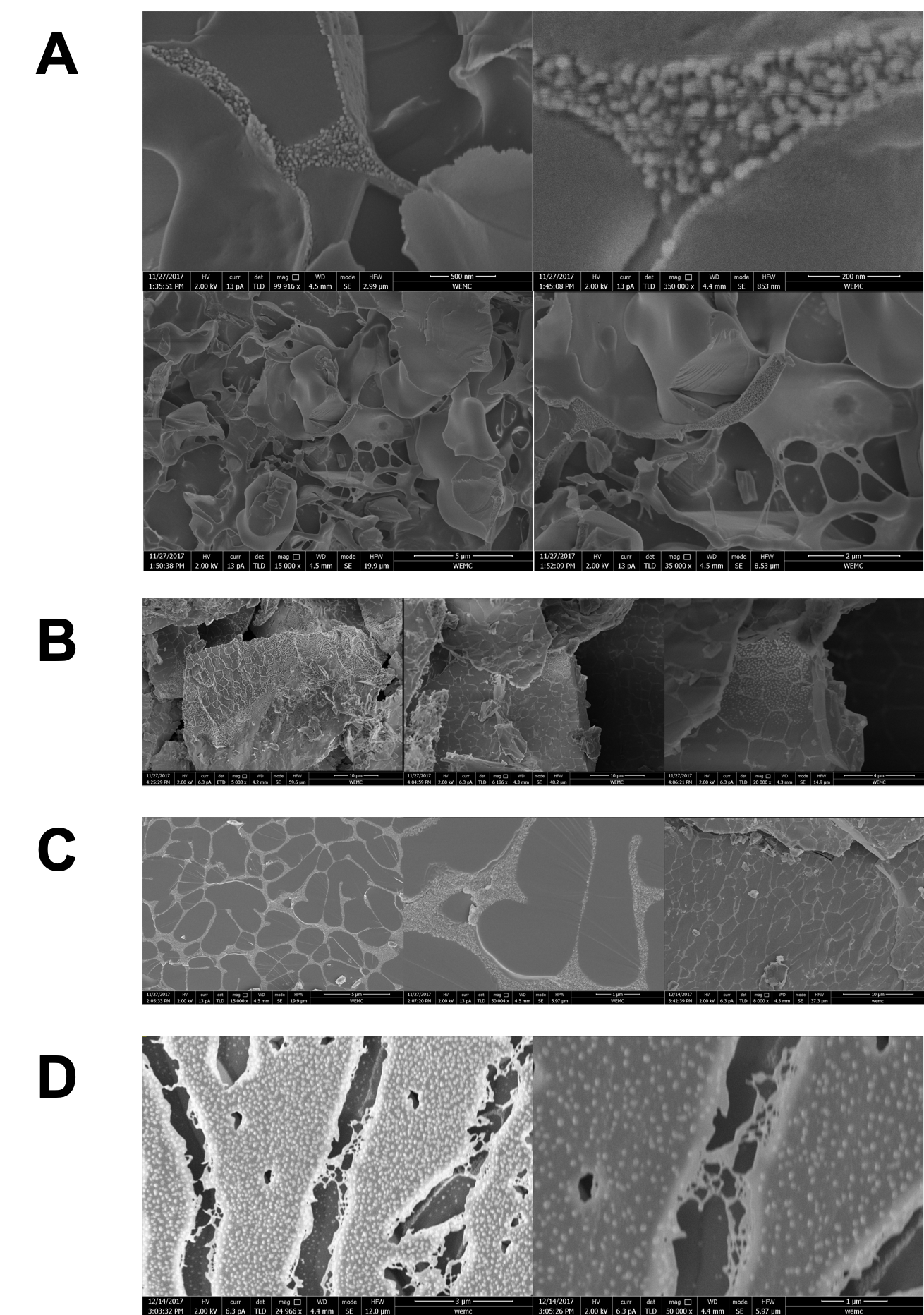

Supplement: S1 Figs — A: Pure protein sample. B: 2% composite sample. C: 5% composite sample. D: 7% composite sample. (TIFF) [file pone.0211059.s001.tiff]
